# Identification of Metabolomic Markers in Frozen or Formalin-Fixed and Paraffin-Embedded Samples of Diffuse Glioma from Adults

**DOI:** 10.3390/ijms242316697

**Published:** 2023-11-24

**Authors:** David Chardin, Lun Jing, Mélanie Chazal-Ngo-Mai, Jean-Marie Guigonis, Valérie Rigau, Catherine Goze, Hugues Duffau, Thierry Virolle, Thierry Pourcher, Fanny Burel-Vandenbos

**Affiliations:** 1 Laboratory Transporter in Imaging and Radiotherapy in Oncology (TIRO), Direction de la Recherche Fondamentale (DRF), Institut des Sciences du Vivant Frederic Joliot, Commissariat a l’Energie Atomique et aux Energies Alternatives (CEA), Université Cote d’Azur (UCA), 06000 Nice, France; david.chardin@nice.unicancer.fr (D.C.); lun.jing@roullier.com (L.J.); jean-marie.guigonis@univ-cotedazur.fr (J.-M.G.); thierry.pourcher@univ-cotedazur.fr (T.P.); 2 Service de Médecine Nucléaire, Centre Antoine Lacassagne, Université Cote d’Azur, 06000 Nice, France; 3 Department of Pathology, University Hospital of Nice, 06000 Nice, France; m.chazal@medipath.fr; 4 Department of Pathology and Oncobiology, Institute for Neurosciences of Montpellier, INSERM U1051, University Hospital of Montpellier, 34000 Montpellier, France; v-rigau@chu-montpellier.fr; 5 Laboratory of Solid Tumors Biology, Institute for Neurosciences of Montpellier, INSERM U1051, University Hospital of Montpellier, 34000 Montpellier, France; c-goze@chu-montpellier.fr; 6 Neurosurgery Department, Institute for Neurosciences of Montpellier, INSERM U1051, University Hospital of Montpellier, 34000 Montpellier, France; h-duffau@chu-montpellier.fr; 7 Team INSERM “Cancer Stem Cell Plasticity and Functional Intra-Tumor Heterogeneity”, Institut de Biologie Valrose, Université Côte D’Azur, CNRS, INSERM, 06000 Nice, France; thierry.virolle@univ-cotedazur.fr; 8 Laboratory “Cancer Stem Cell Plasticity and Functional Intra-Tumor Heterogeneity”, UMR CNRS 7277-UMR INSERM 1091, Institute of Biology Valrose, University Côte d’Azur, 06000 Nice, France

**Keywords:** classification, formalin-fixed and paraffin-embedded tumors, gliomas, metabolomics

## Abstract

**Simple Summary:**

Diffuse gliomas (DGs) are classified according to several histomolecular criteria, including the gliomagenesis pathway, based on *IDH* mutational status. The aim of our retrospective study was to identify the metabolomic signatures of the gliomagenesis pathway and grade of DG by using an untargeted metabolomic technique and to evaluate their diagnostic performances on tumor samples that are formalin-fixed and paraffin-embedded (FFPE). We identified three metabolites, including two novel metabolites of interest in DG that enable prediction of *IDH* mutational status or grade in FFPE samples. We showed that untargeted metabolomics technique may be performed on FFPE samples and be a useful tool for research studies on large cohorts.

**Abstract:**

The aim of this study was to identify metabolomic signatures associated with the gliomagenesis pathway (*IDH*-mutant or *IDH*-wt) and tumor grade of diffuse gliomas (DGs) according to the 2021 WHO classification on frozen samples and to evaluate the diagnostic performances of these signatures in tumor samples that are formalin-fixed and paraffin-embedded (FFPE). An untargeted metabolomic study was performed using liquid chromatography/mass spectrometry on a cohort of 213 DG samples. Logistic regression with LASSO penalization was used on the frozen samples to build classification models in order to identify *IDH*-mutant vs. *IDH*-wildtype DG and high-grade vs low-grade DG samples. 2-Hydroxyglutarate (2HG) was a metabolite of interest to predict *IDH* mutational status and aminoadipic acid (AAA) and guanidinoacetic acid (GAA) were significantly associated with grade. The diagnostic performances of the models were 82.6% AUC, 70.6% sensitivity and 80.4% specificity for 2HG to predict *IDH* status and 84.7% AUC, 78.1% sensitivity and 73.4% specificity for AAA and GAA to predict grade from FFPE samples. Thus, this study showed that AAA and GAA are two novel metabolites of interest in DG and that metabolomic data can be useful in the classification of DG, both in frozen and FFPE samples.

## 1. Introduction

Genetic alterations are one of the paramount mechanisms of carcinogenesis, with metabolic reprogramming being one of their main consequences. Metabolite changes in a tumor can be studied via metabolomic studies [1,2,3], meaning approaches based on determining the levels of different small molecules or metabolites in biological samples (tissue, cells, serum, urine, etc.) are clinically useful. Different metabolomic approaches exist: targeted, based on identifying preselected metabolites within a sample, and untargeted, detecting as many metabolites as possible in a sample. The most common metabolomic techniques used in cancer research are nuclear magnetic resonance (NMR) spectroscopy and mass spectrometry with liquid chromatography (LC-MS) or gas chromatography (GC-MS). Untargeted approaches are of increasing interest because they enable the assessment of thousands of metabolites using a single analysis and can therefore be used to discover novel biomarkers of cancer. Furthermore, the generated data may be used for supervised classification methods, which are emerging tools that help classify tumors along with histological assessment. To be convenient and useful in medical practice, techniques must be adapted to the most common type of tumor sample conditioning and should be retrospectively applicable for each collected sample. In research, most metabolomic studies have been performed on frozen tissues or biological fluids. However, in routine practice within pathology laboratories, representative frozen samples are inconsistently available, whereas tissue samples from all patients are formalin-fixed and paraffin-embedded (FFPE) and then conserved in archives. FFPE samples have a very large potential for exploitable tissues and more and more techniques are adapted for use on these types of specimens. Thus, adapting metabolomic techniques to FFPE samples offers a promising avenue for their application in medical practice. Surprisingly, however, only a small number of metabolomic studies have been performed on FFPE samples [4,5].

Diffuse gliomas (DGs) are the most frequent primary malignant tumors of the central nervous system (CNS) [6]. Because of their highly invasive behavior in the brain and relative radio- and chemoresistance, these tumors remain incurable despite combinations of surgery and adjuvant therapies. Until 2016, DGs in adults were only classified by morphological features as oligodendroglial or astrocytic tumors. The grading system was also based on histological criteria, with the highest grade being grade IV and grade II and grade III characterizing “lower-grade” DGs. In actuality, DGs are genetically heterogeneous tumors. Revisions of the World Health Organization (WHO) classification of CNS tumors in 2016 [7] and then again in 2021 [8] included molecular data in the diagnosis and grading of DGs, leading to an integrative histomolecular diagnosis and increasing the reproducibility of the diagnosis among pathologists. Thus, DG in adults must be classified according to the gliomagenesis pathway, i.e., the presence or absence of mutations in the *IDH* (isocitrate dehydrogenase) genes (*IDH1* or *IDH2*). *IDH*-mutant DGs are separated into oligodendrogliomas (grade 2 or 3, the presence of a 1p/19q codeletion being mandatory for diagnosis) and *IDH*-mutant astrocytomas (grade 2, 3 or 4) with most “lower-grade” DGs belonging to this category. *IDH*-wildtype (*IDH*-wt) DGs are more common, glioblastoma (GBM) being the main representant of this group. GBMs are grade 4 DGs and are associated with the poorest prognosis amongst gliomas. Usually they display high-grade histological features (necrosis and/or microvascular proliferation). Only a small proportion of morphologically lower-grade astrocytomas (i.e., without necrosis and microvascular proliferation) are *IDH*-wt. Because these astrocytomas tend to show a dismal prognosis, they are now classified as GBMs in the 2021 WHO classification [8,9].

Mutations in a gene coding for the enzymes *IDH1* or *IDH2* induces an aberrant enzymatic activity, leading to the production of an oncometabolite, 2-hydroxyglutarate (2HG) [10]. Therefore, *IDH*-mutant DGs are enriched in 2-HG compared with *IDH*-wt DGs [10]. Most metabolomic studies in DG have been performed on fresh or frozen tissues or liquids [1,2,3,11], often in small cohorts [12], and were carried out before the revised version of the WHO classification in 2016 [1,2,3]. Therefore, most data resulting from these studies need to be updated regarding the more recent WHO classification. Few metabolomic studies have been performed on FFPE diffuse gliomas [13]. Using a targeted metabolomic approach, Sahm et al. [13] demonstrated that 2-HG was detectable in FFPE gliomas and, as expected, was significantly increased in *IDH*-mutant DG.

By applying an untargeted metabolomic approach on a large cohort of frozen and FFPE diffuse glioma samples, we aimed to identify the metabolomic signatures associated with the gliomagenesis pathway (*IDH*-mutant or *IDH*-wt) and grade, according to the 2021 WHO classification, from frozen samples, as well as evaluate the diagnostic performances of these signatures in FFPE samples.

## 2. Results

The cohort consisted of 213 tumor samples: 82 pairs of frozen and FFPE tumor samples, along with 44 distinct FFPE samples and 5 distinct frozen samples. The classification of tumor samples is detailed in Table 1. Because most previous metabolomics studies were performed on fresh or frozen tissues and because previous works on FFPE samples have reported a potential wash out of polar molecules after formalin fixation [14], the frozen samples were used as a training set and the FFPE samples were used as a testing set.

All samples were analyzed by LC-MS/MS. A total of 3267 individual peaks were analyzed in the frozen samples, among which 919 could correspond to metabolites from the HMDB database. A total of 2616 individual peaks were analyzed in the FFPE samples, among which 1119 could correspond to metabolites from the HMDB database. A total of 905 distinct metabolites were found in both the frozen samples and the FFPE samples.

### 2.1. Metabolomic Markers of the Gliomagenesis Pathway: IDH-Mutant versus IDH-Wild-Type Tumors

As a first step, metabolomic profiles were compared between *IDH*-mutant gliomas and *IDH*-wt gliomas regardless of histological subtype and grade in order to identify which metabolites were preferentially produced in each molecular pathway.

Thirty-five metabolites were selected (Supplementary File S1), among which only 2HG ([M-H2O+H]^+^ adduct) was selected in more than 85% of the 100 resamples (selection probability of 100%). The mean AUC of the 100 models was 96.8%.

**Figure 1:**
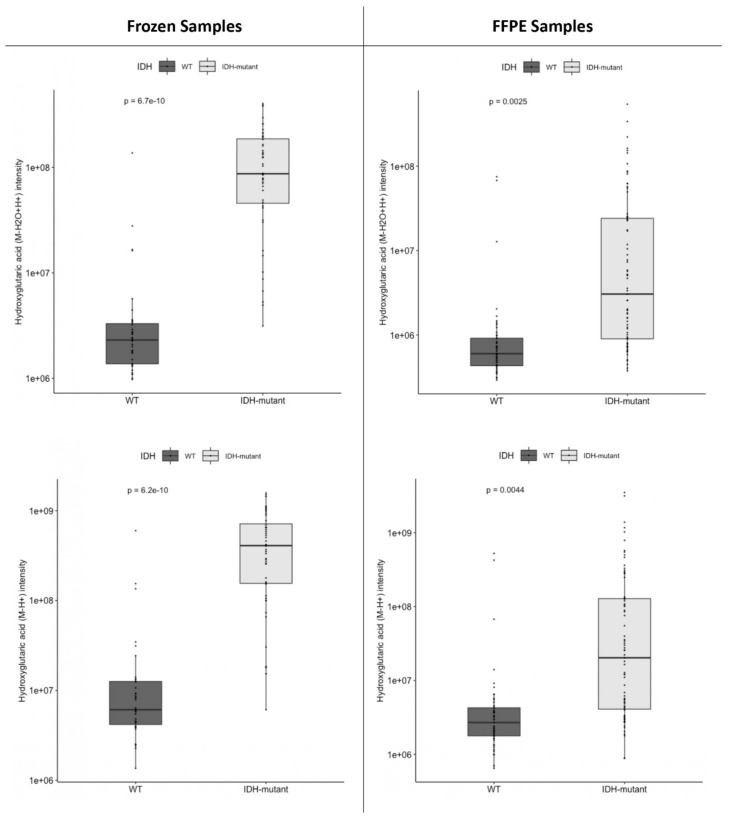
Boxplots of 2-hydroxyglutarate (2HG) levels in *IDH*-wt and *IDH*-mutant glial tumors in either frozen or formalin-fixed and paraffin-embedded (FFPE) tumor samples. (**Top**) 2HG [M-H2O+H]^+^ adduct levels in *IDH*-wt and *IDH*-mutant glial tumors. (**Bottom**) 2HG [M-H]^−^ adduct levels in *IDH*-wt and *IDH*-mutant glial tumors. Scale is logarithmic. *p*-values are given for a Student’s *t*-test.

As shown in Figure 1, the mean values of 2HG [M-H2O+H]^+^ and [M-H]^−^ adducts were significantly higher in the *IDH*-mutant samples than in the *IDH*-wt samples, both in the frozen sample cohort (*p* < 0.001) and in the FFPE sample cohort (*p* = 0.0025 and 0.0044, respectively).

The identification of 2HG was validated both in MS (mass difference of 1.55 ppm) and MS/MS (78.1% match rate) (Supplementary File S2).

**Figure 2:**
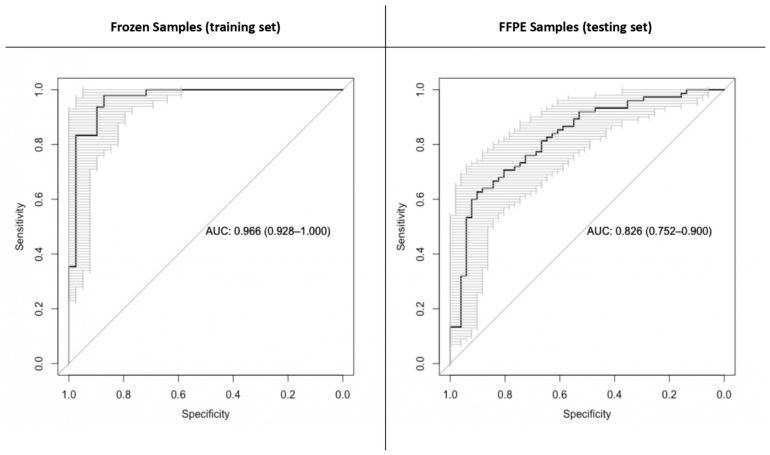
Receiver operating characteristic (ROC) curves for the logistic regression model predicting *IDH* mutational status based on 2-hydroxyglutarate [M-H2O+H]^+^. ROC curves concerning the training set (frozen samples on the left) and the testing set (formalin-fixed and paraffin-embedded samples on the right).

The accuracy with which *IDH* mutation could be predicted using 2HG ([M-H2O+H]^+^ adduct) values was measured using logistic regression and four-fold cross validation on the frozen tissue data. This analysis revealed a mean AUC of 96.6%, a mean accuracy of 94.3%, a mean sensitivity of 98.0% and a mean specificity of 90.4%. When using the 2HG [M-H]^−^ adduct, the same analysis revealed an AUC of 95.5%, an accuracy of 94.3%, a sensitivity of 95.8% and a specificity of 92.2% (Figure 2).

**Figure 3:**
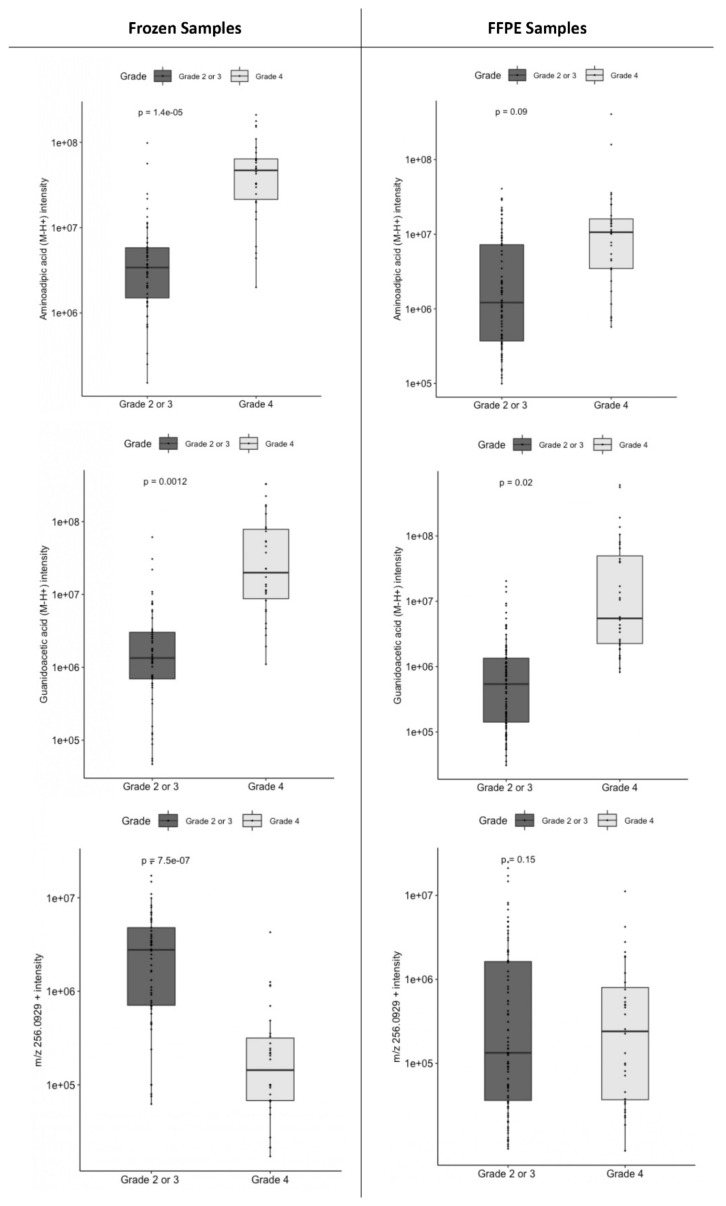
Boxplots of relevant metabolites in lower-grade (2 or 3) and high-grade (4) glial tumors in frozen or formalin-fixed and paraffin-embedded (FFPE) tumor samples. (**Top**) Aminoadipic acid (AAA) levels in *IDH*-wt and *IDH*-mutant glial tumors. Middle. Guanidinoacetic acid (GAA) levels in lower-grade (2 or 3) and high-grade (4) glial tumors. (**Bottom**) Levels of an unidentified peak of *m*/*z* value 256.0929+ in lower-grade (2 or 3) and high-grade (4) glial tumors. Scale is logarithmic. *p*-values are given for a Student’s *t*-test.

When training a logistic regression model using the 2HG [M-H2O+H]^+^ adduct values of the frozen samples and predicting the *IDH* mutational status of the FFPE samples, the AUC was 82.6%, accuracy was 74.6%, sensitivity was 70.6% and specificity was 80.4% (Figure 3). When using the 2HG [M-H]^−^ adduct, the AUC was 80.7%, accuracy was 74.6%, sensitivity was 74.6% and specificity was 74.6%.

When training and testing the model using four-fold cross validation on FFPE samples (2HG [M-H2O+H]^+^ adduct), the AUC was 82.6%, accuracy was 77.8%, sensitivity was 73.8% and specificity was 83.6%.

### 2.2. Metabolomic Markers of Grade: Grade 4 versus Lower Grades

As a second step, grade 4 tumors were compared with lower grades in order to determine which metabolites were associated with the highest grade.

### 2.3. A First Analysis Included Both IDH-Mutant and IDH-Wild-Type Tumors

Thirty-eight metabolites were selected (Supplementary File S3A), among which three metabolites were selected in more than 85% of the 100 resamples (Supplementary File S3A): aminoadipic acid (AAA) (selection probability of 100%), a peak of unidentified positive ion at *m*/*z* 256.0929+ (selection probability of 100%) and guanidinoacetic acid (GAA) (selection probability of 99%). The mean AUC of the 100 models was 97%.

As shown in Figure 3, among the three most selected metabolites, only AAA and GAA had similar differential expressions between grade 4 samples and lower-grade glioma samples both in frozen samples and in FFPE samples. Furthermore, AAA and GAA identification was validated in MS (mass differences of 0.89 ppm and 3.64 ppm, respectively) and in MS/MS (match rates of 70.6 and 69%, respectively); however, the peak of the unidentified ion at *m*/*z* 256.0929+ could not be identified (Supplementary File S2). For this reason, only AAA and GAA were kept for further model training and testing. 

**Figure 4:**
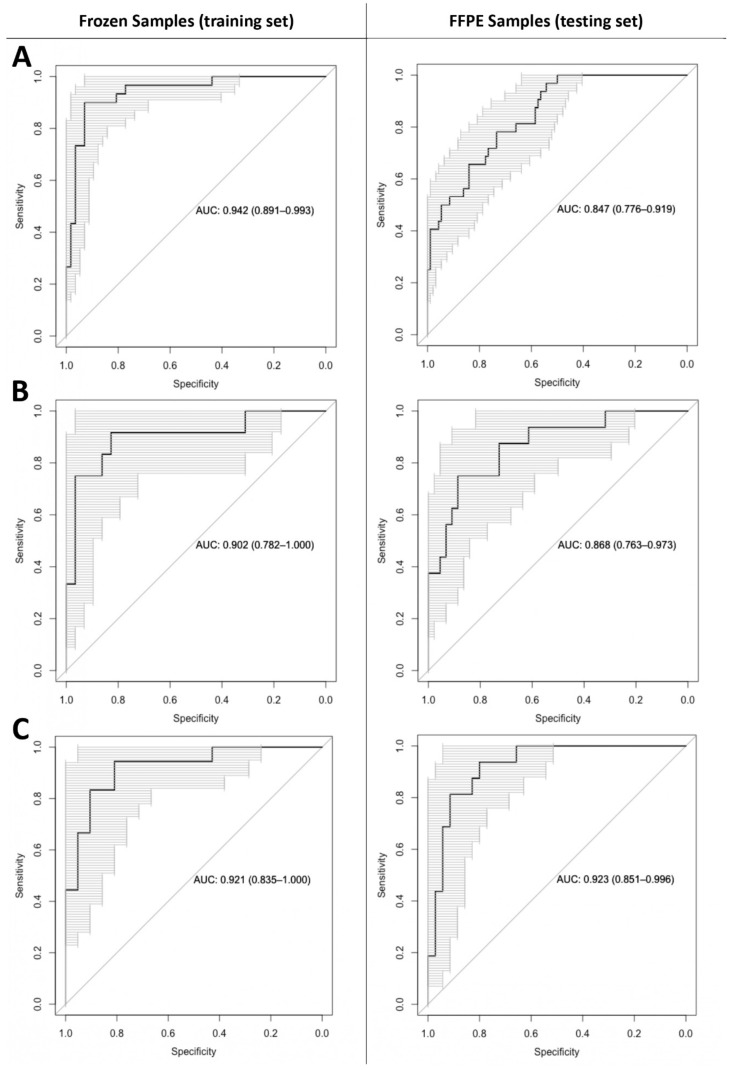
Receiver operating characteristic (ROC) curves concerning predictive models for the classification of glial tumors. All models are trained on frozen samples, ROC curves are then generated concerning the training set (frozen samples on the left) and the testing set (formalin-fixed and paraffin-embedded samples on the right). (**A**) ROC curves for the logistic regression model predicting the histological grades for all glial tumors, regardless of *IDH* mutational status, based on aminoadipic acid (AAA) and guanidinoacetic acid (GAA) levels. (**B**) ROC curves for the logistic regression model predicting histological grade for *IDH*-mutant astrocytomas based on AAA levels. (**C**) ROC curves for the logistic regression model predicting histological grade *IDH*-wt tumors based on GAA levels.

The accuracy with which grade 4 gliomas could be identified using AAA and GAA values was measured using logistic regression and four-fold cross validation on frozen tissue data. This analysis revealed a mean AUC of 94.3%, a mean accuracy of 92.0%, a mean sensitivity of 94.6% and a mean specificity of 90.8% (Figure 4A).

When training a logistic regression model on frozen samples and predicting the grade of FFPE samples, the AUC was 84.7%, accuracy was 74.6%, sensitivity was 78.1% and specificity was 73.4% (Figure 3).

When training and testing the model using four-fold cross validation on FFPE samples, the AUC was 89%, accuracy was 84.1%, sensitivity was 87.0% and specificity was 83.1%

### 2.4. In an Additional Step, IDH-Mutant and IDH-Wild-Type Astrocytomas Were Separately Analyzed

#### 2.4.1. Concerning *IDH*-Mutant Astrocytomas

The metabolite selection step led to the selection of 21 metabolites (Supplementary File S3B), among which 2 metabolites were selected in more than 85% of the 100 resamples: AAA (selection probability of 100%) and the previously described peak of an unidentified positive ion at *m*/*z* value 256.0929+ (selection probability of 100%). The mean AUC of the 100 models was 93.4%.

Only AAA had similar differential expression between *IDH*-mutant grade 4 astrocytomas and *IDH*-mutant low-grade astrocytomas in both the frozen samples and the FFPE samples. This difference was statistically significant in frozen samples (*p* = 0.0084) but not in FFPE samples (*p* = 0.1).

The accuracy with which *IDH*-mutant grade 4 astrocytomas could be identified using the AAA values was measured using logistic regression and four-fold cross validation on frozen tissue data. This analysis revealed a mean AUC of 90.2%, a mean accuracy of 90.4%, a mean sensitivity of 91.7% and a mean specificity of 89.6% (Figure 4B).

When training a logistic regression model on frozen samples and predicting the grade of FFPE samples, the AUC was 86.8%, accuracy was 85.0%, sensitivity was 75.0% and specificity was 88.6% (Figure 4B).

#### 2.4.2. Concerning *IDH*-Wild-Type Astrocytomas

Although all *IDH*-wt astrocytic DGs are now considered as grade 4 DGs, we were interested in the metabolomic differences between morphologically lower-grade (2 or 3) *IDH*-wt astrocytomas and glioblastomas. The metabolite selection step led to the selection of 24 metabolites (Supplementary File S3C), among which 8 metabolites were selected in more than 85% of the 100 resamples: GAA (selection probability of 100%), the previously described unidentified positive ion at *m*/*z* 256.0929+ (selection probability of 100%) and 6 other unidentified ions (selection probability between 86 and 97%). The mean AUC of the 100 models was 89.8%. GAA had the highest weight (Supplementary File S3C), was the only metabolite with similar differential expression between glioblastomas and *IDH*-wt lower-grade astrocytomas both in frozen samples and in FFPE samples (Figure 3) and was the only metabolite that could be validated by MS and MS/MS analysis.

The accuracy with which glioblastomas and *IDH*-wt morphologically lower-grade astrocytomas could be identified using GAA values was measured using logistic regression and four-fold cross validation on frozen tissue data. This analysis revealed a mean AUC of 92.1%, a mean accuracy of 89.7%, a mean sensitivity of 88.8% and a mean specificity of 90.8% (Figure 4C).

When training a logistic regression model on frozen samples and predicting the grade of FFPE samples, the AUC was 92.3%, accuracy was 88.2%, sensitivity was 81.3% and specificity was 91.4% (Figure 4C).

## 3. Discussion

Metabolomic data reflect the consequences of phenotypic and genetic variations in tumors. Diffuse gliomas are a phenotypically and genetically heterogeneous group of tumors for which WHO classification was revised in 2016 and 2021. The revisions impose updates to metabolomic data that have been previously identified according to new classification criteria. In our study, we performed an untargeted metabolomic analysis on a large cohort of diffuse gliomas classified according to the 2021 WHO classification, identified a small selection of metabolites of interest in frozen samples and showed that these metabolites could be detected and used to perform classification in FFPE samples.

To the best of our knowledge, this is the first study using a non-targeted metabolomic approach on FFPE samples of gliomas. Only one previous study confirmed, as expected, that 2HG was overexpressed in FFPE samples of *IDH*-mutant gliomas using a targeted analysis [13]. As anticipated, we found that the metabolomic analysis of FFPE samples was less accurate than that of frozen samples. This was most likely due, at least in part, to the fixation and dehydration steps required in FFPE sample preparation, which may alter the metabolites present. Indeed, as Dannhorn et al. have shown, formalin fixation induces a significant washout of polar molecules [14]. However, this analysis was sufficient to detect and quantify 2HG, AAA and GAA in FFPE samples as well as to use these results to classify these tumor samples with specificities and sensitivities over 70%. Thus, our findings support previous results by Arima et al. suggesting that informative data of metabolic profiles can be retrieved from FFPE tissue materials [15]. Moreover, working with FFPE samples could offer important advantages. Indeed, FFPE samples are the most available material in routine practice. Hence, working with them can offer the advantages of larger cohorts, leading to more statistical power, and also the inclusion of rare entities for which frozen tissues are not always available, such as morphologically low-grade diffuse *IDH*-wt gliomas.

When performing metabolomic studies, statistical procedure plays a very important role in the analysis of the results. Many machine learning methods have been used in this setting [16,17,18]. In our study, we chose to use the widely known LASSO penalized logistic regression along with bootstrapping to select a small number of metabolites of interest. LASSO penalization reduces the risk of overfitting by assuming sparse solutions, leading to simpler models based on a few key features (in this case metabolites). The use of bootstrapping enabled the estimation of selection probability for each metabolite. Focusing on a small number of key metabolites also offers the advantage of fewer false discoveries. Indeed, metabolomic data include a high number of redundant and noisy features and LASSO penalized logistic regression is robust to these unwanted features [19]. In our study, this method led to the selection of a very small number of relevant metabolites in the frozen samples, for which the predictive values could be confirmed in the FFPE samples, confirming the absence of overfitting.

Using an untargeted metabolomic analysis offers both the possibility to verify the presence of known biomarkers and the possibility to identify novel biomarkers. In this study, we found both known and novel biomarkers that revealed the gliomagenesis pathway and tumor grade.

Concerning the gliomagenesis pathway (i.e., *IDH*-mutant status), 2HG was, as expected, significantly overexpressed in *IDH*-mutant gliomas. As only one study has previously shown this [13], we confirm that this biomarker shows a high sensitivity and a high specificity in both frozen and FFPE samples. Interestingly, we found that the [M-H2O+H]^+^ adduct of 2HG had a slightly better diagnostic power than the [M-H]^−^ adduct in FFPE samples. This [M-H2O+H]^+^ adduct of 2-HG could be confused with the [M+H]^+^ adduct of glutaconic acid because both have the same *m/z*. However, since we found the [M+H]^+^ adduct of 2-HG at the same retention time, the forementioned ion should indeed be the [M-H2O+H]^+^ adduct of 2-HG. This adduct could be an alternative to the [M-H]^−^ adduct for the detection of *IDH* mutation if, for instance, performing a single LC-MS analysis in positive ionization mode. 

Even though our statistical analysis led to the selection of multiple metabolites to classify IDH-mutant vs *IDH*-wt tumors, only 2-HG was systematically selected after 100 resampled LASSO regressions (selection probability of 100%). The second most selected metabolite was propionyl-carnithine, but this metabolite had a selection probability of only 48%, which was far lower than that of 2-HG. Thus, there is a high risk that propionyl-carnithine was associated with *IDH* mutation only by chance in our database.

Concerning grade, we found two main metabolites of interest: aminoadipic acid (AAA) and guanidinoacetic acid (GAA). Overall, we found that a logistic regression model based on these two metabolites could predict the grade of glioma samples with an accuracy of 93.1% for frozen samples and 74% for FFPE samples. Interestingly, AAA was more relevant in *IDH*-mutant astrocytomas, for which it could be used to predict the grade with an accuracy of 95.2% in frozen samples and 85% in FFPE samples, whereas GAA was more relevant in *IDH*-wt astrocytomas, for which GAA overexpression was significantly associated with the classical form of *IDH*-wt glioblastoma (grade 4), with an accuracy of 97.2% in frozen samples and 88.2% in FFPE samples.

AAA is a product of the lysine catabolism [20]. The association between AAA and glioblastomas has been poorly described as of yet, but AAA is an emergent metabolite in the medical metabolomic literature [21]. In the study of Locasale et al. [22], AAA was significantly overexpressed in the cerebrospinal fluid of patients suffering from high-grade gliomas as compared with controls without any malignancy. Using ^1^H NMR spectroscopy, Rosi et al. [23] showed that AAA was a marker of glioblastoma stem cell aggressiveness, such cells being correlated with a dismal prognosis. As in our study, Bjorblom et al. [24] found that AAA was increased in *IDH*-mutant glioblastomas as compared with lower-grade *IDH*-mutant gliomas. AAA was also associated with high-grade gliomas in the study of Gorynska et al. [25]. A study on 95 frozen samples of prostatic adenocarcinomas [26] showed that AAA was the only metabolite whose amount was significantly associated with the TNM status and Gleason grade. These studies as well as our own support the hypothesis that AAA is correlated with tumor aggressiveness. The effects of AAA accumulation in the brain are not yet understood. AAA is known to be a gliotoxin [27] and has been shown to induce oxidative stress in the brains of rats [28].

We report an association between guanidinoacetic acid (GAA) levels and tumor grade in gliomas. This finding is consistent with the recent findings of elevated GAA levels in high-grade gliomas reported by Riviere-Cazaux C et al. [29] GAA, also known as glycocyamine or betacyamine, is a metabolite involved in the metabolism of arginine and glycine. GAA is metabolized in creatine via an enzymatic reaction catalyzed by guanidinoacetic acid methyl transferase (GAMT). GAMT is also involved in the de novo synthesis of GAA. Here, we found that GAA was significantly increased in grade 4 gliomas as compared with lower grades. One known cause of elevated GAA in cancers is GAMT overexpression. This overexpression has been shown to be associated with high aggressiveness in sarcomas and carcinomas [30,31,32]. In their study of metastatic pancreatic adenocarcinomas, Yang J et al. [33] showed that GAA overexpression was associated with a more aggressive phenotype (liver metastasis, promotion of cell migration and epithelial–mesenchymal transition) through transcription-activating histone modifications. In light of these data, our observation of significantly increased GAA levels in grade 4 gliomas as compared with lower grades appears relevant but will need to be verified and explained in future studies, in addition to the potential involvement of GAMT in the GAA overexpression. Lastly, we found that GAA was differentially expressed in *IDH*-wt DGs, i.e., between “classical” *IDH*-wt glioblastomas and the morphologically lower-grade *IDH*-wt astrocytomas. Our results suggest that, although these morphologically low-grade tumors are now assimilated to glioblastomas, they are metabolically distinct, at least in the expression of GAA.

Hence, we have shown that 2HG, AAA and GAA are metabolites of interest for the classification of glial tumors. It would be of particular interest to detect these metabolites in vivo using proton magnetic resonance spectroscopy (1H-MRS). As 2HG is a known biomarker of *IDH* mutation, several previous studies have been performed to evaluate its detectability with 1H-MRS. Significant technical limitations remain and 2HG 1H-MRS is not widely used at present [34,35,36]. However, magnetic resonance spectroscopy of 2HG is rapidly improving and could soon be used in routine practice [37,38]. To our knowledge, only very few studies have evaluated the detectability and usefulness of AAA using 1H-MRS in glial tumors. Righi et al. recently identified AAA in a glioblastoma in vivo using 1H-MRS [39]. However, they report that AAA and 2HG cannot be distinguished by one-dimensional 1H-MRS alone but only with two-dimensional techniques such as TOCSY. We did not find any studies concerning GAA 1H-MRS in glial tumors. However, Ensenauer et al. detected GAA using 1H-MRS in the brain of a patient with GAMT deficiency [40]. Even though GAA levels may be higher in the brains of patients with GAMT deficiency than in glial tumors, it would be of interest to study the possible association between GAA levels as assessed by 1H-MRS and glial tumor grades.

We recognize some limitations to our study, most of which are associated with the non-targeted metabolomic approach. Indeed, although these techniques have raised interest in the last decade, the assessments using these techniques show different limitations. The initial raw data include a very high number of peaks of distinct retention times and mass/charge ratios. Working with such high dimensional dataset induces a risk of false discovery. To limit this risk as much as possible, we used different methods:

1. We filtered the initial raw data, removing peaks of lower intensities. This step aims to denoise the data but could also discard metabolites of interest with low concentrations.
2. We used a constrained statistical method, the LASSO penalized logistic regression, which is robust to correlated features and to overfitting.
3. We systematically verified the identification of the selected ions by comparing the obtained mass/charge ratios with mass spectrometry databases and by comparing the corresponding MS/MS spectrums to spectrum databases.
4. We verified the results obtained using the frozen sample cohort using the FFPE sample cohort, which we used as a validation dataset.

Technical variations represent another limitation of the untargeted metabolomic techniques because they can induce batch effects. To reduce the putative batch effect in our study, we performed the LC-MS/MS analysis of the frozen samples in a single continuous batch with a minimal number of columns. Afterwards, we performed the analysis of FFPE samples in a second batch. Here, we focus our study on the metabolites found in both the frozen and FFPE samples, therefore the metabolites were identified in two different batches of LC-MS/MS analyses.

The age of the studied samples could have an impact on our results. Indeed, Isberg et al. [41] observed a linear decrease in the mean signal intensities for the lower mass range (100–500 *m*/*z*) over a period spanning 70 years of sample age. In our study, the samples were collected between 2005 and 2020. Therefore, the oldest samples were 15 years old and were essentially represented in the groups of rarest types (oligodendrogliomas, *IDH* mutant astrocytomas and morphologically lower-grade *IDH*-wt astrocytomas). In their study, Isberg et al. found normalized intensities of smaller metabolites to linearly decrease over time, with a weak gradient of 0.218. These results suggest that there might be a difference of a factor of 1.3 in the ion intensities between the samples collected in 2020 and the samples collected in 2005. This should not affect the validity of our results since the differences observed in ion intensities between groups are closer to a factor 10. However, this could lower the chances of selecting metabolites with smaller variations between groups.

Furthermore, our untargeted approach was only semiquantitative. We could not use stable isotopes of the highlighted metabolites because they were not known prior to the analysis and therefore a possible matrix effect, affecting the quantification of peak intensities, cannot be corrected. However, the total ionic currents in all analyses remained quasi-constant, comforting the validity of the measured intensities. Nonetheless, further targeted studies including stable isotopes could lead to results with higher precision.

Finally, even though it would have been interesting to explore the possible metabolomic variations associated with the histological sub-types of *IDH*-mutant gliomas, we could not perform this analysis because we did not include sufficient oligodendrogliomas to be statistically relevant.

In conclusion, we have shown that the [M-H]^−^ and [M-H2O+H]^+^ adducts of 2HG could be detected and used to determine *IDH* mutational status both in frozen and FFPE samples and that aminoadipic acid and guanidinoacetic acid could be detected and used to evaluate the grade of astrocytomas both in frozen and FFPE samples.

Furthermore, untargeted metabolomic studies can be performed on FFPE samples of adult diffuse gliomas and could represent promising tools to further understand the heterogeneity of these tumors and to develop related supervised classification methods.

## 4. Materials and Methods

### 4.1. Sample Collection

Frozen and FFPE tumor samples were collected from the Pathology Department of Nice University Hospital and from the platform CRB-CHUM of Montpellier University Hospital (Biobank BB-0033-0031). Tissue collections were declared to the French Health Ministry (as required by French legislation). Written consent and/or non-opposition was obtained for each patient. All cases were classified according to the 2021 WHO classification of CNS tumors by two expert neuropathologists (FBV and VR). Of note, all the morphologically “lower-grade” *IDH*-wt astrocytomas harbored one of the following molecular features of GBM: association of chromosome 7 gain and chromosome 10 loss, *EGFR* amplification and/or a mutation of the *TERT* promoter.

### 4.2. Sample Preparation

Sample preparation was performed by an expert technician with 15 years of experience. Frozen tissues (sections of 100 µm thickness obtained from 5 mm^3^ samples) were placed in microcentrifuge tubes and ground in 1 mL of cold methanol (LC-MS grade, Merck Millipore, Molsheim, France) using pestles. FFPE tissues (sections of 100 µm thickness obtained from 5 mm^3^ samples) were mixed with 1 mL of methanol at 70 °C for 30 min and then at 0 °C for 15 min and centrifuged at 13,000 rpm for 10 min at 0 °C. The supernatant and homogenized frozen tissues were incubated overnight at −20 °C and then centrifuged at 15,000 rpm for 15 min. The supernatant was then removed and dried using a SpeedVac concentrator (SVC100H, SAVANT, Thermo Fisher Scientific, Villebon-sur-Yvette, France). Lyophilized samples were resuspended in 100 µL of a 50:50 acetonitrile-H2O mix (LC-MS grade, Merck, Millipore, Burlington, MA, USA) prior to LC-MS/MS analyses.

### 4.3. LC-MS/MS Analysis

The frozen samples and FFPE samples were analyzed separately.

Metabolomic analyses were performed using LC-MS/MS. Liquid chromatographic analysis was performed using the DIONEX Ultimate 3000 HPLC System (Thermo Fisher Scientific, Waltham, MA, USA). An amount of 10 µL of each sample was injected into a Synergi 4 µm Hydro-RP 80 Å, 250 × 3.0 mm column (Phenomenex, Le Pecq, France). The mobile phases were composed of 0.1% formic acid (Thermo Fisher Scientific, Waltham, MA, USA) in water (A) and 0.1% formic acid in acetonitrile (B). The gradient was set as follows, with a flow rate of 0.9 mL/min: 0% phase B from 0 to 5 min, 0–95% B from 5 to 21 min, holding at 95% B to 21.5 min, 95–0% B from 21.5 to 22 min, holding at 0% B until 25 min for column equilibration. Mass spectrometry analysis was carried out on a Q Exactive Plus Orbitrap mass spectrometer (Thermo Fisher Scientific, Waltham, MA, USA) with a heated electrospray ionization source, HESI II, operating in both positive and negative mode. High-resolution accurate-mass full-scan MS and top 5 MS2 spectra were collected in a data-dependent fashion at resolving powers of 70,000 and 35,000 at *m*/*z* 400, respectively. All samples were successively treated in the same run. 

### 4.4. Metabolomic Profiling

The data from frozen samples and FFPE samples were processed separately.

Raw data obtained from positive and negative ionization mode were analyzed separately using MZmine (version 2.53) [42]. Mass detection was performed using the mass detector tool (mass detector: wavelet transform, MS level 1; noise level 10^5^, scale level: 5, wavelet window size: 30%). Chromatograms were detected using the ADAP chromatogram builder [43] (MS level: 1, minimum group size in number of scans: 5, group intensity threshold: 5 × 10^2^, minimum highest intensity: 10^5^, *m*/*z* tolerance: 10 ppm). Peaks were separated using the peak extender module (M/Z tolerance: 10 ppm, minimum height: 10^5^). Retention times were normalized using the retention time calibration module (*m*/*z* tolerance: 10 ppm; retention time tolerance (relative): 10%, minimum height: 10^5^). Peaks were then aligned using the RANSAC aligner (random sample consensus) algorithm with a tolerance of 10 ppm in *m*/*z* and 1 min in retention time. Peaks were then identified using the Human Metabolome Database [44] (HMDB, version 3.0) with 10 ppm of mass tolerance. Missing values were filled in using the same *m*/*z* and RT range gap filler with a tolerance of 10 ppm in *m*/*z*. The results obtained with each polarity were combined and, for metabolites that were identified in both modes, only the mode for which the peak had the highest mean intensity was considered. Furthermore, only peaks that had intensities over 10^6^ in at least 30 samples were kept for analysis to eliminate noisy peaks. Finally, non-attributed values in the final databases were replaced by an arbitrary small value corresponding to the peak intensity threshold (10^5^).

After statistical analysis, the metabolites of interest were individually verified (MS and MS2 spectra) using Compound Discoverer 3.1 (Thermo Fisher Scientific, Waltham, MA, USA) and matching between the experimental *m*/*z* and the reference MS/MS spectrums of the mzCloud and Metlin databases [45] (available as Supplementary Materials).

### 4.5. Statistical Analyses

Statistical analyses were carried out using R version 3.6.3 with the packages “glmnet”, “pROC” and “Metabonalyze”. Raw data were mean-centered, scaled and log-transformed before performing a logistic regression with L1 penalization (LASSO).

The first step consisted of selecting relevant features using frozen samples as a training set. Logistic regression with LASSO penalization was used with 4-fold cross-validation (CV) to select the optimal value of lambda, for which maximum area under the receiver operating characteristic curve (AUC) was obtained. Using this method, metabolites that contributed the most to the classification between groups were selected and other metabolites were attributed null coefficients. The robustness of each selected metabolite was measured using a resampling-based bootstrap procedure, as performed by Bujak et al. [19] and described by Bach [46]; 100 resamples were performed and the metabolites were ranked according to their selection probability based on the number of times they had been selected (i.e., had non-null coefficients) after 100 resamples. The mean of the performances of the models were measured as a mean AUC. The mean weights of each metabolite were also recorded.

As a second step, logistic regression models were trained using only metabolites for which the selection probability was over 85%. These models were first evaluated on frozen samples using 4-fold cross validation. Then, the reproducibility of the results was evaluated by training a logistic regression model using frozen samples and testing this model using FFPE samples. The performances of the model were calculated as AUC, accuracy, sensitivity and specificity using ROC curve analysis.

The mean values of the metabolites of interest were also compared between groups using Student’s *t*-test.

## Figures and Tables

**Table 1:** Histomolecular classification of the diffuse glioma samples in the study according to the WHO 2021 classification and the WHO 2007 classification.

Histological Subtypes	*IDH* Status	WHO 2021 Grade	WHO 2007 Grade	Frozen Samples *n* = 87 (%)	FFPE Samples *n* = 126 (%)
Oligodendroglioma	Mutant	2	II	7 (8.0%)	15 (11.9%)
Astrocytoma	Mutant	2	II	19 (21.8%)	29 (23.0%)
Astrocytoma	Mutant	3	III	10 (11.5%)	15 (11.9%)
Astrocytoma	Mutant	4	IV	12 (13.8%)	16 (12.7%)
Astrocytoma *	Wild type	4	II	9 (10.3%)	15 (11.9%)
Astrocytoma *	Wild type	4	III	12 (13.8%)	20 (15.9%)
Glioblastoma	Wild type	4	IV	18 (20.7%)	16 (12.7%)

* These morphologically “lower-grade” astrocytomas are classified as glioblastoma in the 2021 WHO classification but have been voluntarily individualized in the study.

## Data Availability

Data are contained within the article and Supplementary Materials.

## Supplementary Materials

The following supporting information can be downloaded at: https://www.mdpi.com/article/10.3390/ijms242316697/s1.
