# Supplementary material for: Identification of Metabolomic Markers in Frozen or Formalin-Fixed and Paraffin-Embedded Samples of Diffuse Glioma from Adults
Source: Int J Mol Sci. 2023 Nov 24;24(23):16697. doi: 10.3390/ijms242316697 (PMC10705927; doi:10.3390/ijms242316697)
Supplement: Supplementary file 1 [file ijms-24-16697-s001.zip › Supplementary NEW/Supplementary file S2.pdf]

2-Hydroxyglutaric Acid

Mass Spectrum 1

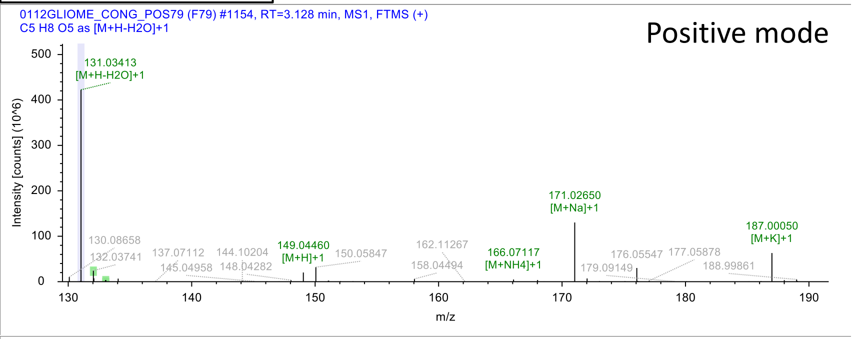

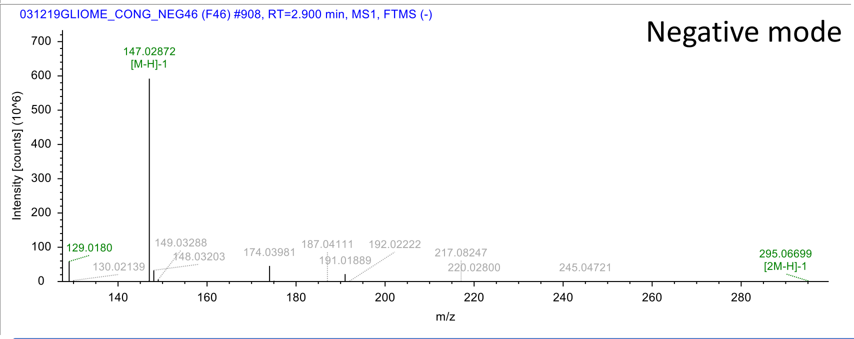

Structure	Name	Formula	Molecular Weight	ΔMass [Da]	ΔMass [ppm]
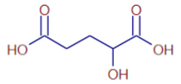	(+/-)-2-Hydroxyglutaric acid	C5 H8 O5	148.03717	0.00023	1.55

Mass Spectrum 2

Experimental spectra

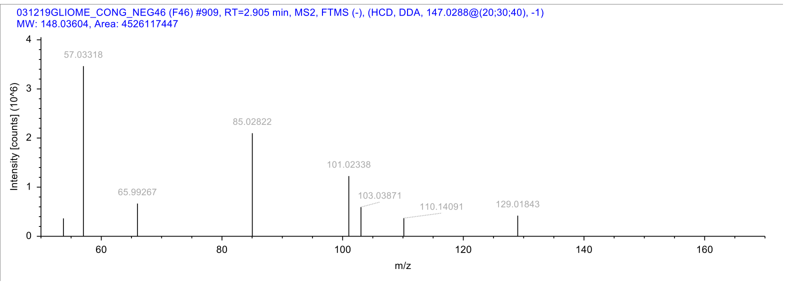

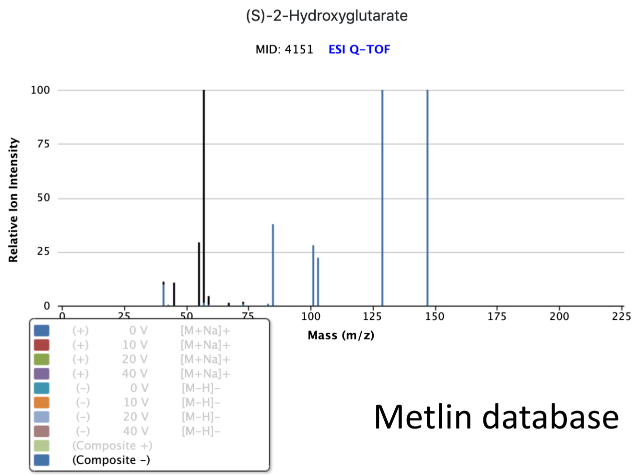

Metlin database

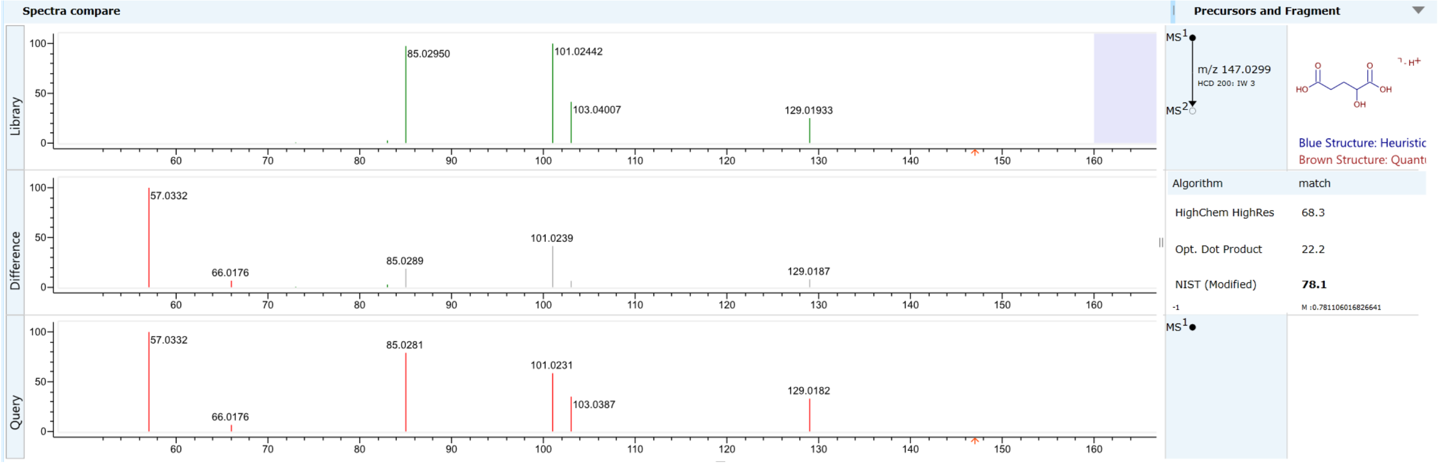

Aminoadipic Acid

Mass Spectrum 1

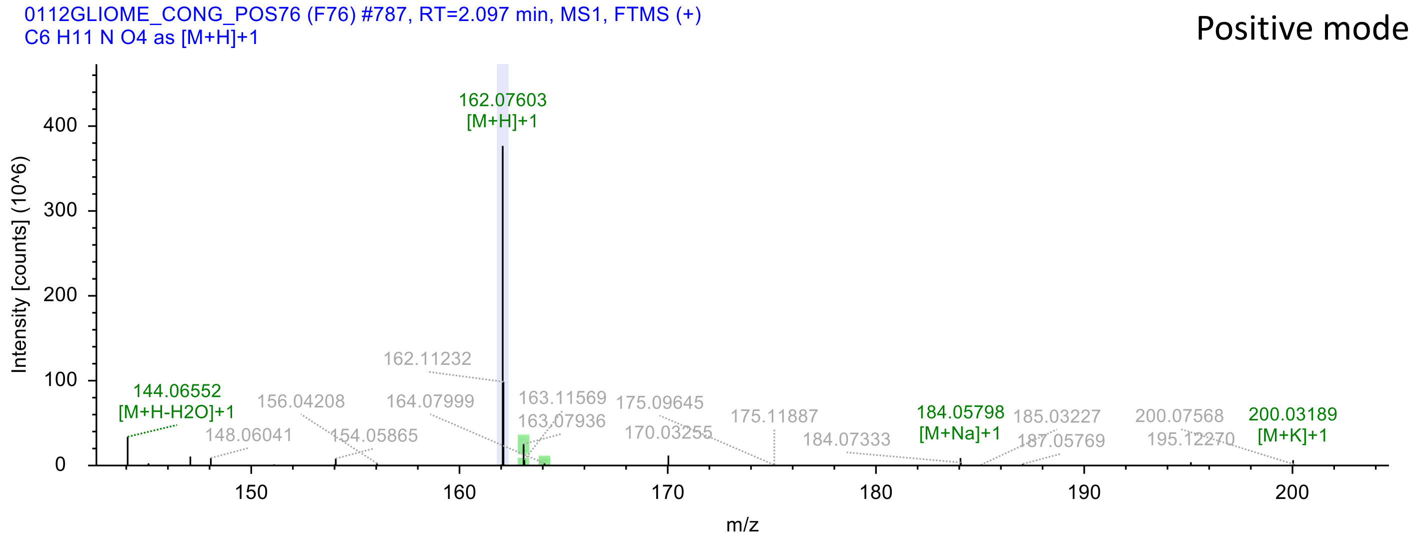

Structure	Name	Formula	Molecular Weight	ΔMass [Da]	ΔMass [ppm]
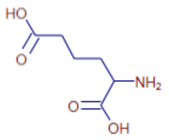	.alpha.-Aminoadipic acid	C6 H11 N O4	161.06881	0.00014	0.89

Mass Spectrum 2

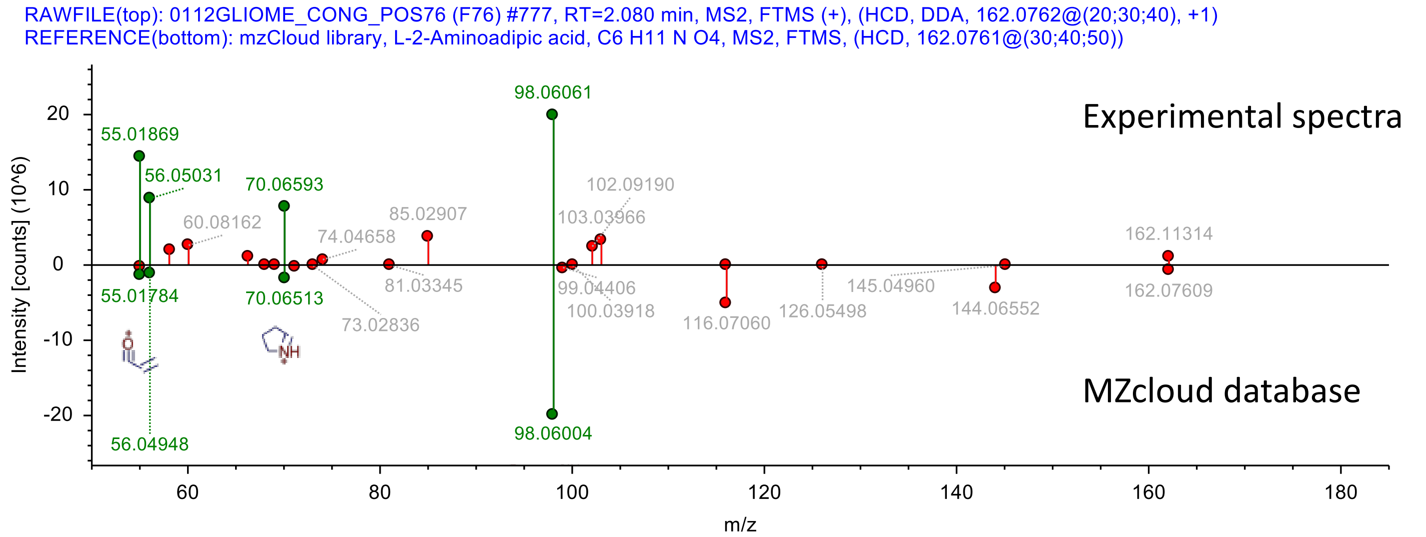

Structure	Name	Formula	Molecular Weight	ΔMass [Da]	ΔMass [ppm]	Match	Best Match	Best Sim. Match	Scan #	Type	mzCloud ID	KEGG ID
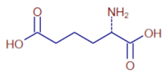	L-2-Aminoadipic acid	C6 H11 N O4	161.06881	0.00014	0.88	70.6	70.6		777	Identity	Reference-3214	C00956

Guanidoacetic Acid

Mass Spectrum 1

Positive mode

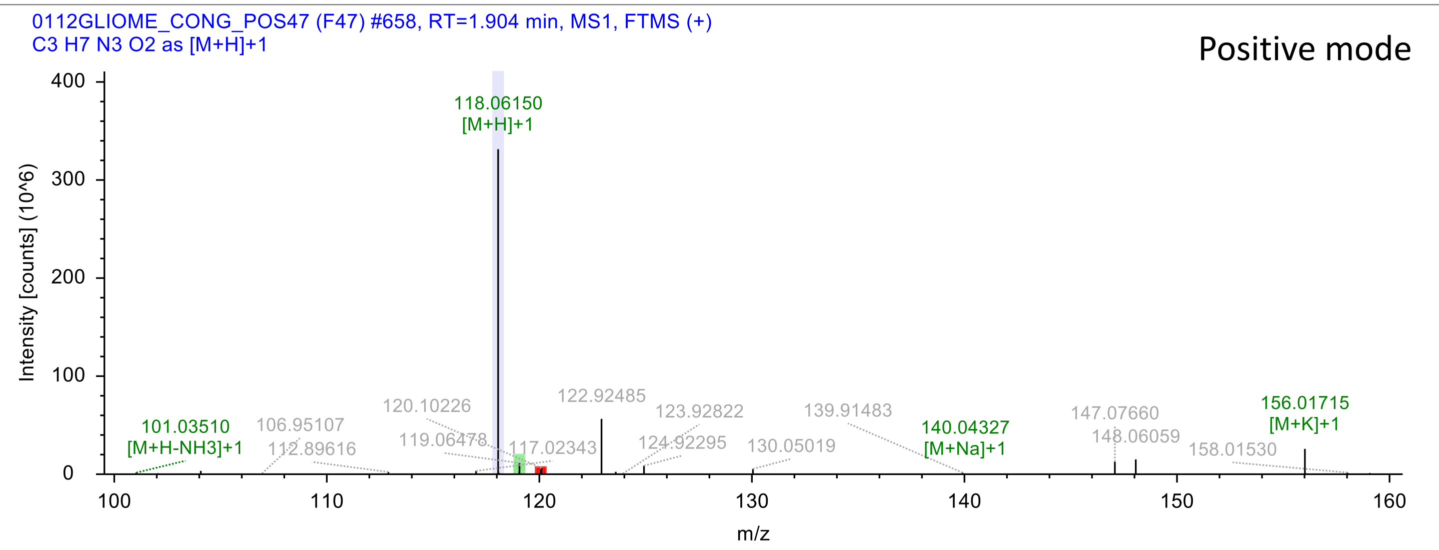

Formula	Molecular Weight	ΔMass [Da]	ΔMass [ppm]
C3 H7 N3 O2	117.05383	0.00043	3.64

Mass Spectrum 2

Experimental spectra

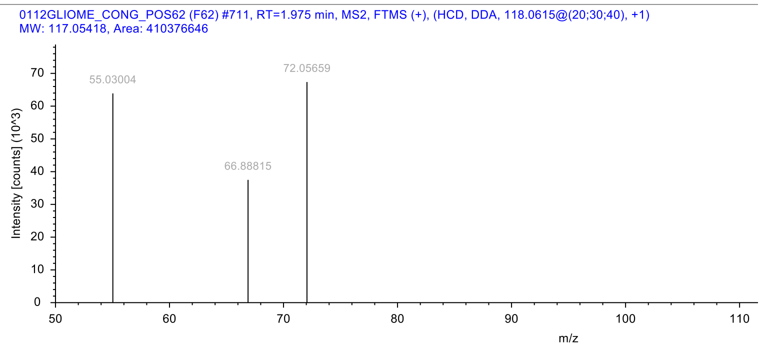

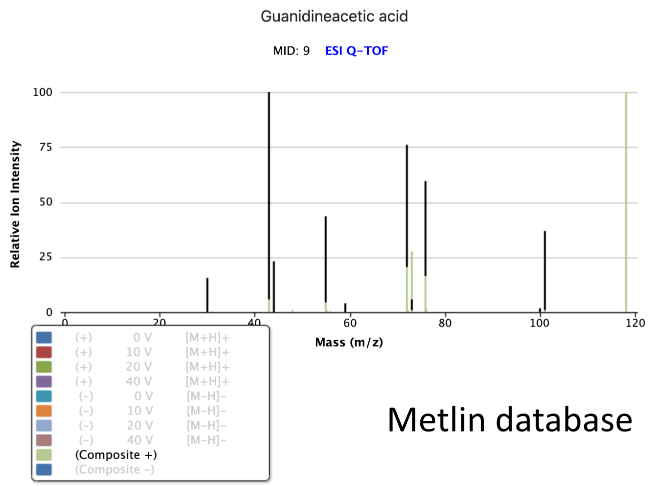

Metlin database

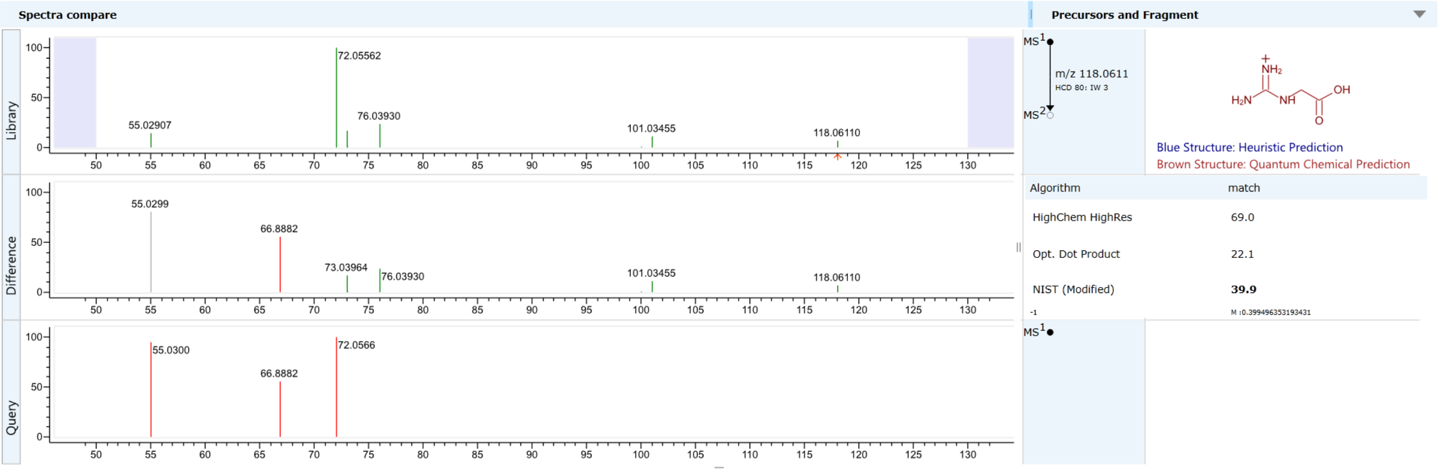

Unidentified m/z 256.09296+ ion

Mass Spectrum 1

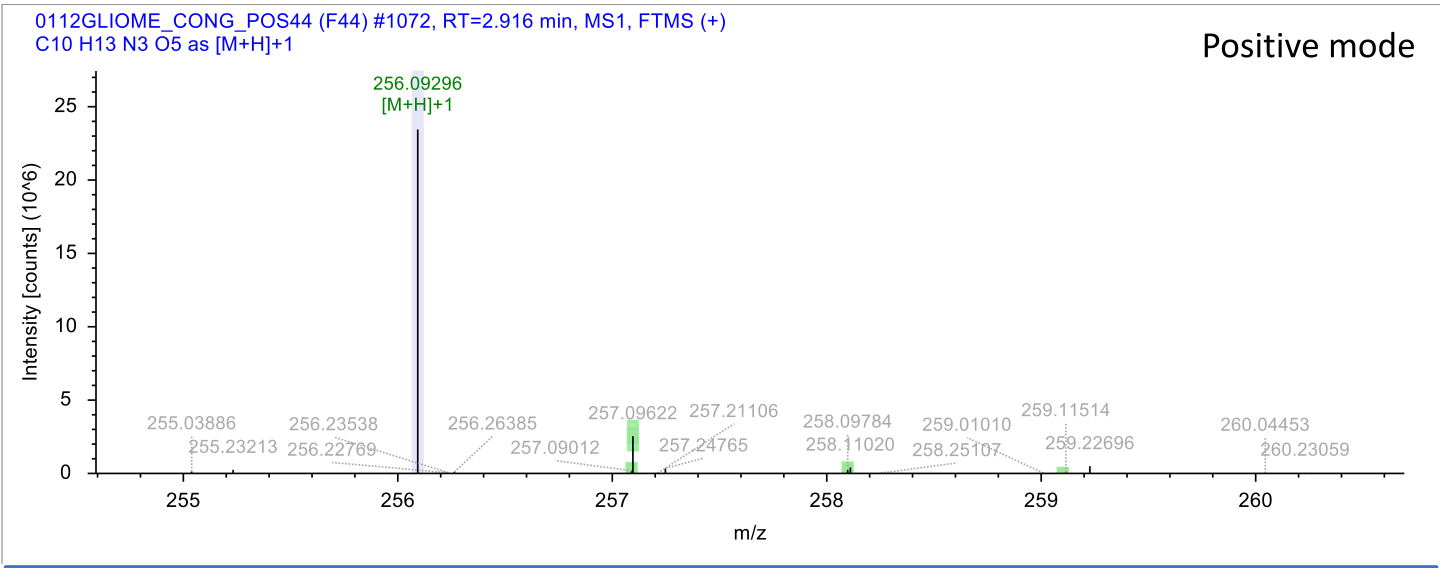

Formula	Molecular Weight	ΔMass [Da]	ΔMass [ppm]	RDBE	H/C	Rank	# Matched Iso.	# Missed Iso.	# Matched Frag.	SFit [%]	Pattern Cov. [%]	MS Cov. [%]	MSMS Cov. [%]
C10 H13 N3 O5	255.08552	0.00004	0.16	6.0	1.3	1	5	0	4	72	100.00	100.00	66.56

Mass Spectrum 2

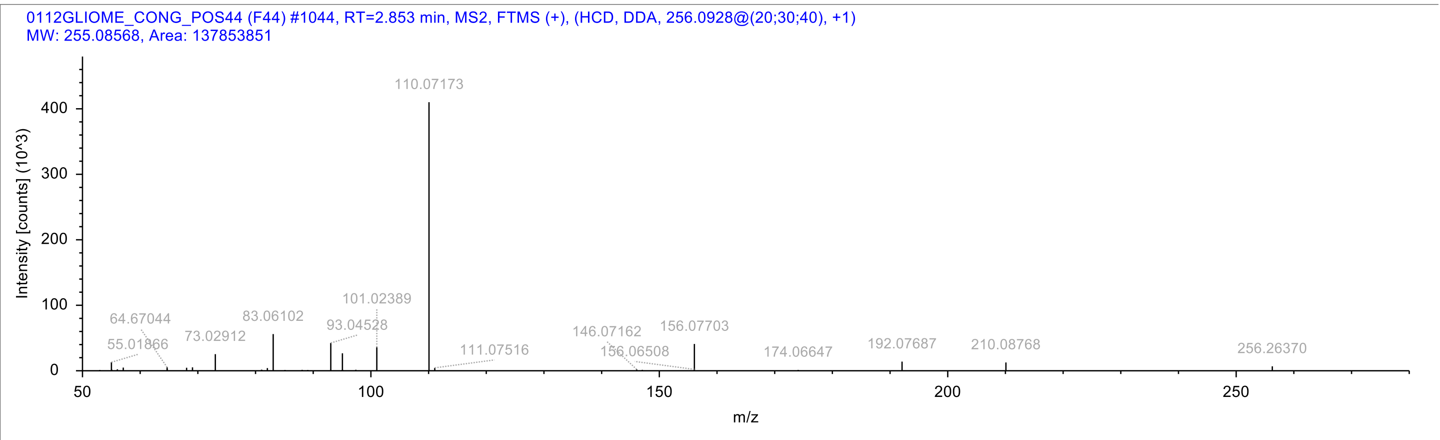

→ No MzCloud or Metlin match
